# Evaluating human health risk assessment tools for contaminated soil: a comparative review

**DOI:** 10.1007/s11356-026-37411-w

**Published:** 2026-02-04

**Authors:** Rusalina Lupu, Diana-Mariana Cocarta, Iason Verginelli

**Affiliations:** 1 Department of Energy Production and Use, Faculty of Energy Engineering, National University of Science and Technology POLITEHNICA Bucharest, Splaiul Independentei No. 313, 006042 Bucharest, Romania; 2 Research Centre for Advanced Materials, Products and Processes/CAMPUS, National University of Science and Technology Politehnica Bucharest, Splaiul Independentei No. 313, Bucharest, Romania; 3 Academy of Romanian Scientists, 030167 Bucharest, Romania; 4 Laboratory of Environmental Engineering, Department of Civil Engineering and Computer Science Engineering, University of Rome “Tor Vergata”, Rome, Italy

**Keywords:** Contaminated sites, Human health risk assessment, Environmental modeling, Software tools

## Abstract

At the European Union level, approximately 60–70% of soil is in unhealthy or degraded conditions. One of the soil threats is the legacy of industrial pollution, as historic industrial pollutants discharged into the ground continue to pose risks to both the environment and human health. As part of the legislative measures adopted in the late 90 s, Human Health Risk Assessment (HHRA) was introduced as a standardized method for evaluating risks associated with contaminated sites. To support the quantification of these risks, various software tool models were developed. This study reviews 12 HHRA tools for contaminated sites developed across different countries. First, an overview of national legislative frameworks concerning contaminated sites, with a particular focus on the use of HHRA as a decision-making tool, is provided. Subsequently, the study compares and discusses the methodologies adopted by each tool, the exposure pathways and receptors considered, the integrated contaminant databases, and additional features provided by the models. The comparison highlights the diversity of functionalities offered by the different tools, reflecting a lack of harmonization among national regulations regarding contaminated site management. Beyond the need for a harmonized approach at the EU level, potential future developments include the design of more user-friendly interfaces capable of expanding exposure scenarios, updating contaminant lists (including emerging pollutants such as PFAS), integrating uncertainty analysis, incorporating Geographic Information System (GIS)-based visualizations, and integrating artificial intelligence (AI)/machine learning (ML).

## Introduction

Soil is an important factor in delivering ecosystem services such as water, food, habitat for different species, or base for constructions. However, in the last decades, soil is in continuous danger due to erosion, floods and landslides, contamination, loss of soil biodiversity and organic matter, salinization (Frelih-Larsen et al. 2017).

The European Green Deal started as an ambitious plan to transform society from fossil fuel switching to solutions oriented toward healthier and less polluting industries. The European Union (EU) aims to achieve climate neutrality by 2050 and rehabilitate biodiversity under the EU Biodiversity Strategy for 2030 (European Commission 2020). Current initiatives are directed toward protecting the environment, reducing climate change, and achieving safe agricultural principles. There are eight areas of interest reflected within the Green Deal, and soil health is being targeted as one of the most important factors (Orgiazzi et al. 2022).

The European Union has started promoting legislative initiatives on soil matters as part of the EU Green Deal. In 2021, it adopted the “EU Soil Strategy” for 2030, which comprises direct measures to protect the soils and further use them in a sustainable manner. As a result, the European Committee (EC) has launched in 2023 a mission dedicated to the restoration of soil health by 2030 entitled “Soil Deal for Europe,” within the Horizon program (Orgiazzi et al. 2022). In addition to strengthening the initiatives, the new European Law dedicated to the soil monitoring framework: “Soil Monitoring Law” (European Commission 2023) was announced in June 2023. It was estimated that around 60–70% of soils in the EU are currently not in good condition (Orgiazzi et al. 2022); herein, these initiatives were set up to reduce the threats and maintain soil health, and to adopt environmentally friendly soil management practices. The magnitude of soil degradation and challenges from warfare impact on soils are widely discussed in the “State of soils in Europe” (Akca et al. 2024). In this context, the management and quality of soils are of essential importance regarding future environmental, agricultural, and climate endeavors. Consequently, the utilization of large-scale monitoring and modeling tools would ensure proper development, implementation, and monitoring of soil-related policies (Orgiazzi et al. 2022).

Following the European strategies on soil health protection and monitoring, countries are in the process of establishing approaches in preserving the soil. As part of soil monitoring, environmental risk assessment investigation is a good approach to identify the contaminated sites, the extent of it, and what future measures are to be implied. What is an environmental risk assessment, and why is it important to conduct one? These are significant questions that were addressed in the framework of the current research paper (European Commission 2021; European Environmental Agency 2022).

Soil pollution can be characterized as the accumulation of persistent toxic compounds, chemicals, salts, radioactive materials, or disease-causing agents in soils, which negatively impact the ecosystems, respectively, contributing to the development of human diseases (Hernandez Soriano 2014). The estimation provided by the World Health Organization (WHO) indicates that environmental hazards account for a substantial proportion of mortality, reaching 23% of all deaths globally, equivalent to 12.6 million individuals in 2012. The deleterious effects of pollution-induced illnesses are disproportionately concentrated among low and middle-income nations, with children, women, and the most vulnerable populations facing this impact (UNEP 2019).

Addressing pollution can effectively reduce the prevalence of diseases, mitigate environmental degradation, enhance the quality of life, particularly that of women and children, mitigate the adverse effects on humans health, and prevent economic losses associated with reduced income and productivity (UNEP 2019; Mathez and Smerdon 2009).

As a result of further monitoring and modeling results, scientists raised a worldwide alarm on changing the way industries work and reduce globally the pollution (Commonwealth of Australia 2012; European Environmental Agency 2021). Based on a recent study developed by FAO, thousands of synthetic and natural compounds and elements are discharged into the environment and can contribute negatively to human health and the environment. The fate of the contaminants in soil (their retention or mobility to other environmental compartments and their impact on living organisms) is determined by the inherent properties of the contaminant as well as the specific soil characteristics of the area (FAO and UNEP 2021b). The investigation of cancer induced a pivotal moment in the subject of risk as it encouraged the perspective of risk scientists and healthcare experts toward the investigation of Health Risk Assessments (HRAs) domain (Theodore and Dupont 2012). On the other hand, monitoring and understanding the contaminant’s effects on human beings is complex work, requiring institutions to have diverse focus groups dedicated to each problem and deliver a solution in a timely manner. The main focus consists of identifying the pollutants of concern and their concentration in the media, pathways of exposure, and sources (ISPRA 2020; FAO and UNEP 2021a).

Based on the above approach, taking into consideration the big amount of data to be processed, countries from the European Union and internationally have adopted and developed environmental tools in order to optimize the datasets, monitor closely the pollution and its effects on human health, afterward proposing a cost-effective option for remediating/cleaning up the contamination (ISPRA 2020).

**Fig. 1:**
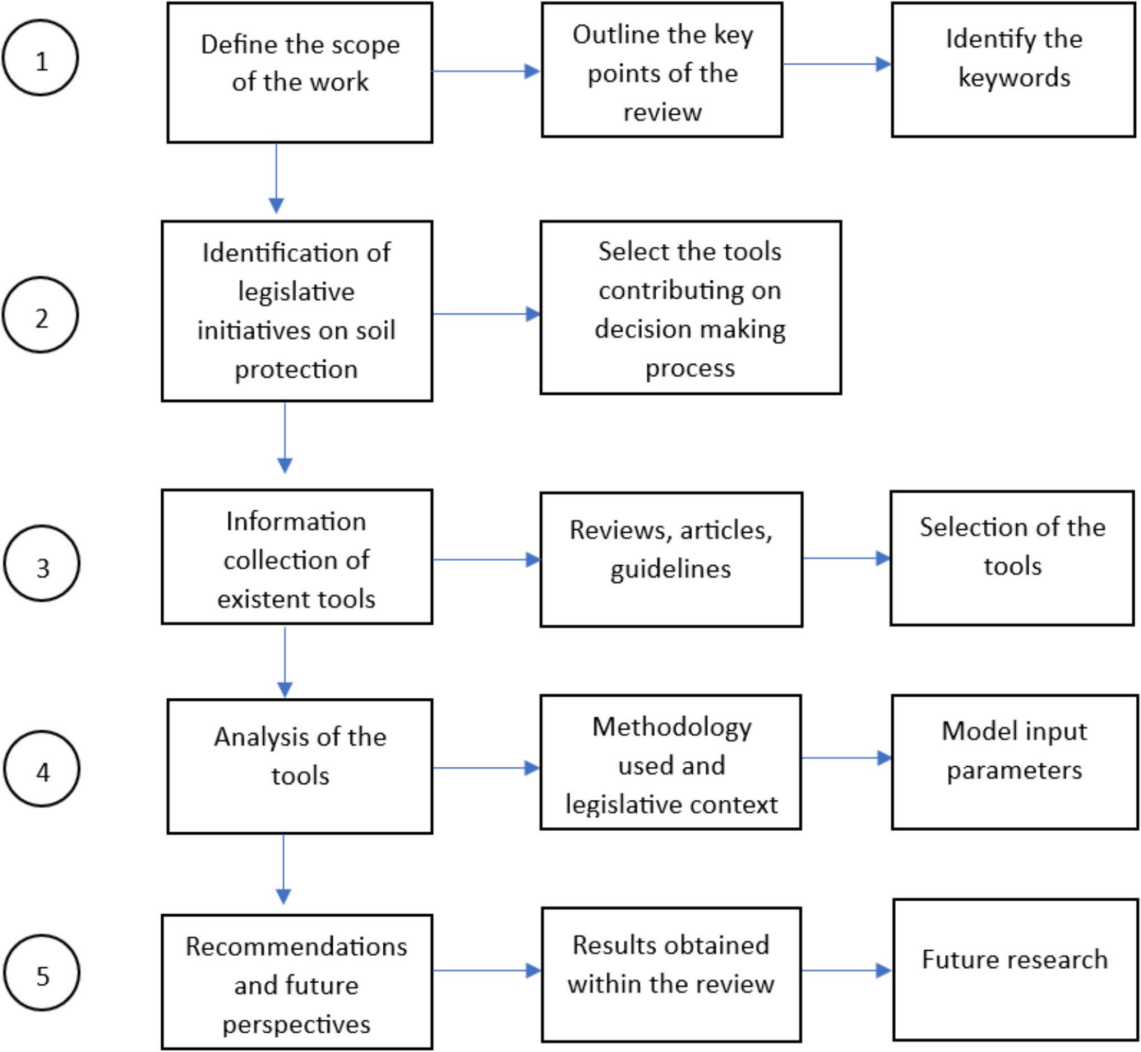
The structure proposed for the current review paper, inspired after Gao and Pishdad-Bozorgi (2019)

In appraising the existent Human Health Risk Assessment (HHRA) tools for contaminated sites available and their applicability in the current soil monitoring legislation at EU level, the present study has adopted a systematic review of existing publications. The scope of the current research paper is focused on providing an up-to-date and comprehensive review of information on the differences and similarities between the human health risk assessment tools implemented and used in different countries. The research methodology was developed in five steps, as per Fig. 1: (i) scope identification, (ii) soil legislative context and methodologies, (iii) data gathering on the 12 selected tools for human health risk assessment, (iv) analysis of the selected tools, and (v) recommendations and future perspectives. The analysis section of the review paper covers the legislative context of soil monitoring (European Commission 2023), exposure pathways, receptors, types of contaminants considered, and data integration within the HHRA tools.

## Research methodology

The information was extracted from Google Scholar, ScienceDirect, Taylor & Francis, MDPI, and Springer Link journal articles. The bibliographic data was gathered as well from sources offered by the international organizations: European Chemical Agency (ECHA), Environmental Protection Agency of the United States (EPA USA), European Environmental Agency (EEA), Joint Research Center (JRC), Institute of Environmental Research and Protection (ISPRA), World Health Organization (WHO). The keywords used in the research strategy were “soil strategy,” “soil monitoring,” “Green Deal,” “environmental risk assessment,” “human health risk assessment,” “risk characterization,” “environmental risk management,” “soil and groundwater contamination tools,” “risk assessment tools,” “HHRA tools,” “management of contaminated sites in Europe,” “management of contaminated sites in USA,” “soil quality legislation,” “uncertainty in HHRA,” and “spatial analysis in risk assessment.”

A systematic review of English sources and limited sources in other languages has been performed (Snyder 2019). Herein, the information presented in the current study may not be complete due to the vast amount of literature availability.

In brief, the current review was focused on analyzing 12 selected software tools that have been studied and evaluated/compared with other software tools via published articles, reviews, and more. Moreover, the selection of the tools was made according to the availability and access to the information online. The review will rely on the research chart diagram proposed in Fig. 1, inspired by the review paper of Gao and Pishdad-Bozorgi (2019).

## Soil pollution and environmental risk assessment as a mechanism for ecosystem preservation

The evaluation of the potential impacts from a chemical, physical, microbiological, or psychosocial hazard on a designated human population or ecological system, under specific conditions and for a given duration, is referred to as *risk assessment* (Commonwealth of Australia 2012). The risk assessment process is generally divided into Ecological Risk Assessment and Human Health Risk Assessment.

Ecological risk assessment evaluates the potential or likelihood of adverse effects resulting from human activities on the ecosystems, including plants, lakes, and animals (Flemström et al. 2004). On the other hand, the United States Environmental Protection Agency (US EPA) defines the human health risk assessment (HHRA) as the “process to assess the harm may be caused to human health due to exposure to contaminants present in the environment” (US EPA 2024a).

In some contexts, human health risk assessment and ecological risk assessment are considered part of the broader concept of Environmental Risk Assessment (Muralikrishna and Manickam 2017). For instance, Ehsan Arzaghi et al. (2020) defines the Environmental Risk Assessment as a systematic approach for assessing and managing the risk associated with human health and ecological entities caused by an event occurring in the environment.

**Fig. 2:**
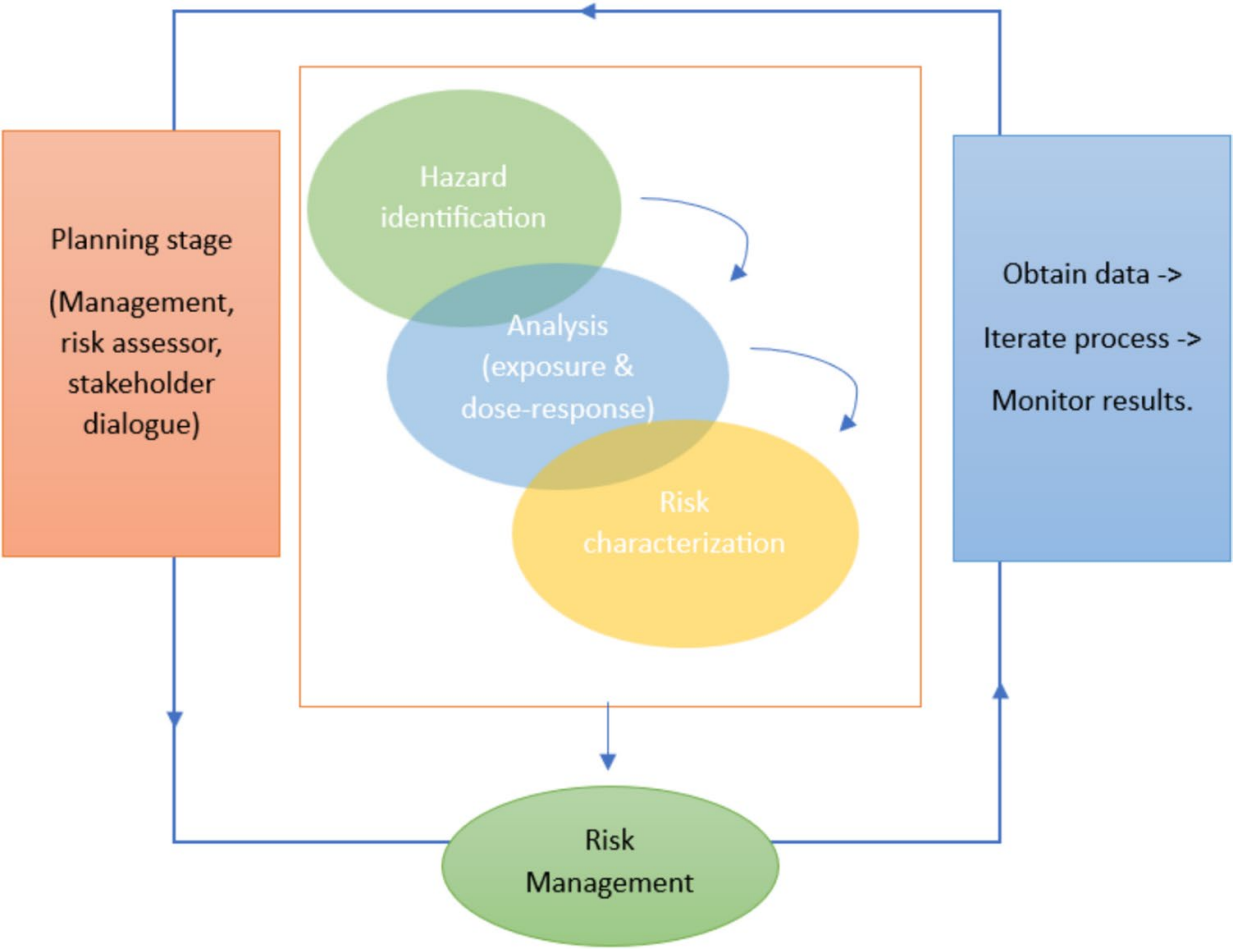
Steps of HHRA adapted after US Epa (2024a, b, c)

The use of risk assessment can be traced back to early ages, (Pepper et al. 2013); the principle was adopted in the process of HHRA, too. It represents a multistep process consisting of hazard identification, exposure assessment, dose–response assessment, and risk characterization (Muralikrishna and Manickam 2017), and finally, followed by results analysis and adoption of risk management targeted at reducing the risk (Theodore and Dupont 2012). The overall stages are adjusted by each individual based on the in-depth output expected from the investigated scenario (Fig. 2) (Norton and Suter 2019).

In short, *hazard identification* within the HHRA represents the identification of the stressors that cause human health effects (e.g., tumors, reproductive defects) and the likelihood of their occurrence (US EPA 2021a, 2022a, b). Next steps, *exposure assessment* consists of determining what contaminant quantity humans were exposed to during a certain timeframe, and how many people were exposed. *Dose–response assessment* encompasses the identification of the associated health problems given the exposure to different contaminants (US EPA 2022a, b; Norton and Suter 2019). The decision-making process on the risk associated with the stressor is known as *risk characterization* (WHO 2021). In practice, each step of the assessment has a short risk characterization described, which includes limitations, assumptions, and uncertainties (US EPA 2022a, b). The whole structure of risk assessment is primarily focused on *risk analysis* and, subsequently, the other two *risk management* and *communication* as part of the study completion (WHO 2008).

### Software tools for risk assessment of contaminated sites

Climate disasters worldwide have brought people’s attention to adapting regulations and laws for preserving natural resources and ecosystem re-establishment. Soil contamination is one of the priority list topics, as it can be noted from the increase of legislation adoption at EU, respectively country level (Zhang et al. 2023). As stated in the review of Khan et al. (2018), the contaminants’ presence in the natural soil environment can lead to micro and macro-scale biotic community disturbances. Therefore, it is critical to understand the risks and effects of the contaminated sites toward the environment. Computational programs in this context are of help to deliver a higher quality and speedy data output. Thus, different countries have developed their own programs and regulations on conducting risk assessments for contaminated soil. In time, the procedures have evolved as part of the technological and research improvements; institutions developed guidelines, handbooks, and model databases based on sound science to conduct human health risk assessments (Zhang et al. 2023; Dong et al. 2015; De Brujin et al. 2003; Jeffries 2009; Fischhoff 2015; US EPA 2022a, b; EFSA 2021; Du et al. 2022).

Online literature review shows the existence, at country levels, of tools dedicated to HHRA of contaminated sites (Mahammedi et al. 2020; Swartjes 2015). However, not all the countries take advantage of fully digitalizing the way of performing an assessment, as in the case of Romania (Government of Romania 2015). The lack of database tools averts the assessment from being performed in a rapid manner. Consequently, the final information is compiled over a prolonged period of time and requires high costs.

Along the time, countries such as the UK have developed tools (e.g., CLEA software) in order to assess the possibility of human exposure to a contaminant, in an easy and practical way (Jeffries 2009; DEFRA 2015). The same situation is in Italy where, due to the presence of numerous industrial contaminated sites (Comba et al. 2011), research institutions (e.g., ISPRA and universities) have elaborated various software programs for risk assessment: ROME developed in 2001, Giuditta in 2006, RACHEL in 2012, Risk-net developed in 2012, and ROME PLUS in 2021 (Bartolozzi et al. 2008; ASTM 2004). The Netherlands authorities in the 1990 s have presented a list of potentially contaminated sites (a number of 110,000) (European Environmental Agency 2000). The CSOIL tool was developed in 1994 in the Netherlands in response to the need to comprehend the complexity of the situation with regard to human health (last update done in 2020) (Breemen et al. 2020). In the USA, the trigger started much earlier, in 1978 with the “Love Canal” case, when the local population discovered their houses were built on a former highly toxic chemical landfill. This event led to the first regulation on soil remediation (Dupuis and Knoepfel 2015). As the need to safeguard the environment and human health has grown over time, the USA has created a variety of programs and tools, such as the RBCA toolkit by GSI Environmental Inc. (Connor et al. 2007, 2001). The Danish Environmental Protection Agency (Danish EPA) in collaboration with the scientists has developed various tools to assess soil contamination; one of them is the JAGG (*Jord*, *Afdampning*, *Gas*, *Grundvand*), developed in 2000 (Danish Environmental Protection Agency 2000) and last updated in 2022 (Danish Environmental Protection Agency 2022), and the GrundRisk Landfill risk assessment tool, developed in 2019 for the transport of contaminants released from landfills (Danish Environmental Protection Agency 2019). In Romania, the first attempt at tackling soil pollution via evaluation of environmental pollution started in 1997 (Environmental Ministry of Romania 1997), but more attention in terms of contaminated sites legislative measures was given later in 2007(Government of Romania 2007). Even if a number of 1682 potentially contaminated and contaminated sites were identified, this number is not yet definitive (Government of Romania 2015). In line with the current regulation in force, the National University of Science and Technology Politehnica of Bucharest (Politehnica Bucharest) developed the RECOLAND tool (2010) and an upgraded version of it, REMPET (in 2015), for internal use only.

It is worth noting that the approach to HHRA tools may vary across different countries, as detailed in subsequent sections which will tackle the methodology and legislation used, and analysis of the tools, as presented in Table 1.

**Table 1:** Summary of HHRA software tools, methodology, specific country of legislation on contaminated sites, and the corresponding gaps

Software Tools	Country developed	Methodology/model used	Legislation or guidance framing the site assessment	Gaps	Reference
(1). CLEA	UK	- Multiple algorithms to calculate soil-to-plant transfer (Environment Agency 2009b); - USEPA approach in accordance with UK data for particulate emission factors - Johnson and Ettinger model to derive the volatilization factor from soil to indoor media - American Society for Testing and Materials (ASTM) standard to calculate volatilization factor from surface soil to ambient	Environmental Protection Act 1990 of UK and related Contaminated-land statutory guidance part 2A	- Based on Chen’s (2018) paper, the generic assessment is overestimating the risk results considering the commercial land use scenario for vapor pathways in the case of some contaminants (ethylbenzene, xylene and naphthalene) - Incorporation of algorithms within the detailed risk assessment to cover a wide range of pathways and contaminants behavior in the media, for a precise risk calculation. As an example, using the allotment scenario, the bioconcentration in fat products (e.g., eggs) is not considered as a factor for potential pollutant linkage, for the contaminants: polychlorinated-p-dioxins (PCDDs) and polychlorinated dibenzofurans (PCDFs) with low solubility and volatility properties (Megson et al. 2011) - Limited confidence in the bioaccessibility of dietary lead, under the selection of Relative Bioavailability for use in lead (Pb) generic assessment criteria (Cocerva et al. 2024)	Jeffries (2009), Jeffries and Martin (2009), USEPA (1996), ASTM (2004), Johnson and Ettinger (1991), Chen (2018), Sun et al. (2020), DEFRA ( 2012), Megson et al. (2011), Cocerva et al. (2024)
(2). CSOIL	Netherlands	The remediation urgency method (RUM) (*SaneringsUrgentie Systematiek*, in Dutch), updated with Circular on soil remediation (VROM, 2006), RIVM Reports	Dutch Soil Protection Act; Soil Remediation Circular Supplement, 2013	- The model does not assess direct drinking water exposure. The groundwater scenario can be used only in the case contaminated groundwater has no effect on the above soil layers and is permeating through the pipeline affecting the potable water (Breemen et al. 2020) - Risk value generation for the plant uptake pathway requires a number of environmental parameters which may not always be available to the user (Takaki et al. 2014)	Breemen et al. (2020), Brand et al. (2006), Swartjes et al. (2012), Takaki et al. (2014), Otte et al. (2001), Van Den Berg (1995), Waitz et al. (1996)
(3). JAGG	Denmark	The model was developed based on “The behavior of chemical substances in soil and groundwater” by the Department of Environmental Technology, Denmark Technical University (DTU) in 1995/1996	Guidance No. 6 & 7 of the Danish Environmental Protection Agency (DEPA): “Guideline for remediation of contaminated sites” 1998	- The model is linked to the Guideline for remediation of contaminated sites by DEPA, herein it is limited to this administrative aspect - Model engine operates on proprietary basis, resulting in diminished transparency and potential of errors - Tool available only in Danish language	Chambon et al. (2011), Dixit et al. (2016), Larsen (2007)
(4). RBCA toolkit	USA	American Society for Testing and Materials (ASTM) Risk-Based Corrective Action (RBCA) standard E-2081–00 and E-1739–95 Fate and transport models adopted in the tool: - Source depletion considering soil and groundwater - ASTM and US EPA outdoor air volatilization - Johnson & Ettinger model - Groundwater Mass Flux indoor air model - Soil leaching model proposed by ASTM - Dual-equilibrium desorption - Point-source air dispersion - Domenico groundwater solute transport, with decay options	The Comprehensive Environmental Response, Compensation and Liability Act (CERCLA), the Resource Conservation and Recovery Act (RCRA), and the US EPA guidelines for human health risk assessment (1989a, 1996)	- Considering the groundwater contamination exposure via leaching from soil, the model assesses the risk as a direct drinking from groundwater. The model has not envisioned the permeation possibility through pipelines (Pinedo et al. 2014) - No on-site indoor dust inhalation pathway is considered in the tool - The model is for commercial use	Connor et al. (2007), Pinedo et al. (2014)
(5). Risk-net	Italy	ASTM risk-based corrective action (RBCA) standard and ISPRA Guidelines	Legislative Decree No. 152/2006 approving the Code on the Environment		Verginelli (2019)
(6). S-RISC	Belgium	– Flemish–Leach model “DAEB methodology on risk evaluation and risk-based background values (2011)” – -US-EPA (1996b), part 2 – -RBCA – -Volasoil model	- Decree on soil remediation and soil protection - Ordinance on the management and clean-up of soils - Decree on the management of soils	- An isolated layer of contaminants such as: pure product, floating or sinking layer in groundwater is not included in the model - The model is for commercial use	Spence and Walden (2013); Schollaert et al. (2011); Van Keer (2019)
(7). RISC5 or BP RISC	USA, UK	ASTM RBCA standard, US EPA’s Risk Assessment Guidance for Superfund (RAGS) and Johnson and Ettinger (J&E) models	Similar to the RBCA Toolkit and CLEA countries of provenience: - The Comprehensive Environmental Response, Compensation and Liability Act (CERCLA) - The Resource Conservation and Recovery Act (RCRA) - The US EPA guidelines for human health risk assessment (1989a, 1996) - The Environmental Protection Act 1990 of UK and related Contaminated-land statutory guidance part 2A	- No on-site indoor dust inhalation pathway is considered in the tool - No outdoor vapor inhalation pathway considered in the tool - The last version upgrade was released in 2010 - The tool is commercial	Spence and Walden (2013)
(8). CalTOX	USA	The US Environmental Protection Agency (Epa 1989; Federal Register, 1992), the US EPA risk assessment guidance for superfund (RAGS) manual, and the California Department of Toxic Substances Control (DTSC) standards	Similar to the RBCA Toolkit country of provenience: - The Comprehensive Environmental Response, Compensation and Liability Act (CERCLA) - Resource Conservation and Recovery Act (RCRA) - US EPA guidelines for human health risk assessment (1989a, 1996)	- The software has not considered surfactants and volatile metals. It can be used for partially ionized organic chemicals - The tool cannot be used in the case the water occupies more than 10% of the soil area investigated, also is working only for low concentrations of contaminants. In the case of concentration exceedance of the solubility limit, the modeling results are not considered valid. Can be used as a complement while conducting detailed transport/transformation/exposure assessments - The model is for commercial use	Schwalen and Kiefer (1996), McKone TE and Lawrence Livermore National Lab CA (1993), McKone (1993a, McKone 1993b), McKone (1993c, a, b)
(9). RECOLAND, REMPET*	Romania	US EPA standards	Romanian legislation, No. 756/1997 on contaminated sites	- Do not cover the inhalation or groundwater drinking exposure pathways - The tool considers only carcinogenic compounds - The hazard index (HI) is not represented - The tool has not been validated against other implemented tools and limited case studies	Dumitrescu, et al. (2012), Jeffries (2009)
(10). CARO PLUS	Germany	The ASTM standard 2004, the Multimedia Environmental Pollutant Assessment System (MEPAS) developed by Strenge and Smith in 2006, the Volatilization model, and the AUATOX ecosystem model	The Soil Contamination Act of 1999 and amended in 2006	- Tool intended for megasites - Assessment of quantitative pathways only for organic pollutants derived from dense nonaqueous phase liquids (DNAPL) and light non-aqueous phase liquid (LNAPL)	McKnight and Finkel (2013), Mcknight et al. (2010), EUGRIS (2003), Mcknight et al. (2015)
(11). ROME PLUS	Italy	“Procedure for the evaluation and use of soil gas data in the risk assessment of contaminated sites” (Linee Guida SNPA 17/2018), the “Design of vapor monitoring in contaminated sites” (Appendix B), and the “Active Flux Chambers monitoring” (Linee Guida SNPA 15/2018)	Legislative Decree No. 152/2006 approving the Code on the Environment	- The number of chemical compounds available for risk assessment is provided in the ISS-INAIL database 2018 - Last updated in 2021 as v1.1. version - The risk analysis targets soil-gas and emission flux chamber assessments only	ISPRA (2021)
(12). K-RBCA	South Korea	The ASTM standard guide for risk-based corrective action (ASTM 2004) and the US EPA guidance (US EPA 1991a, 1992, 1997)	Soil Environment Conservation Act 1995	- The tool allows the assessment of Tier 1 and partially Tier 2 risk assessment, which uses basic and conservative values. The results tend to be overestimated - Human health risk assessment can be calculated for the on-site exposure scenarios only - It targets the Korean soil contamination guidelines, and the tool is available in Korean language only	Nam et al. (2011a, b)

**Fig. 3:**
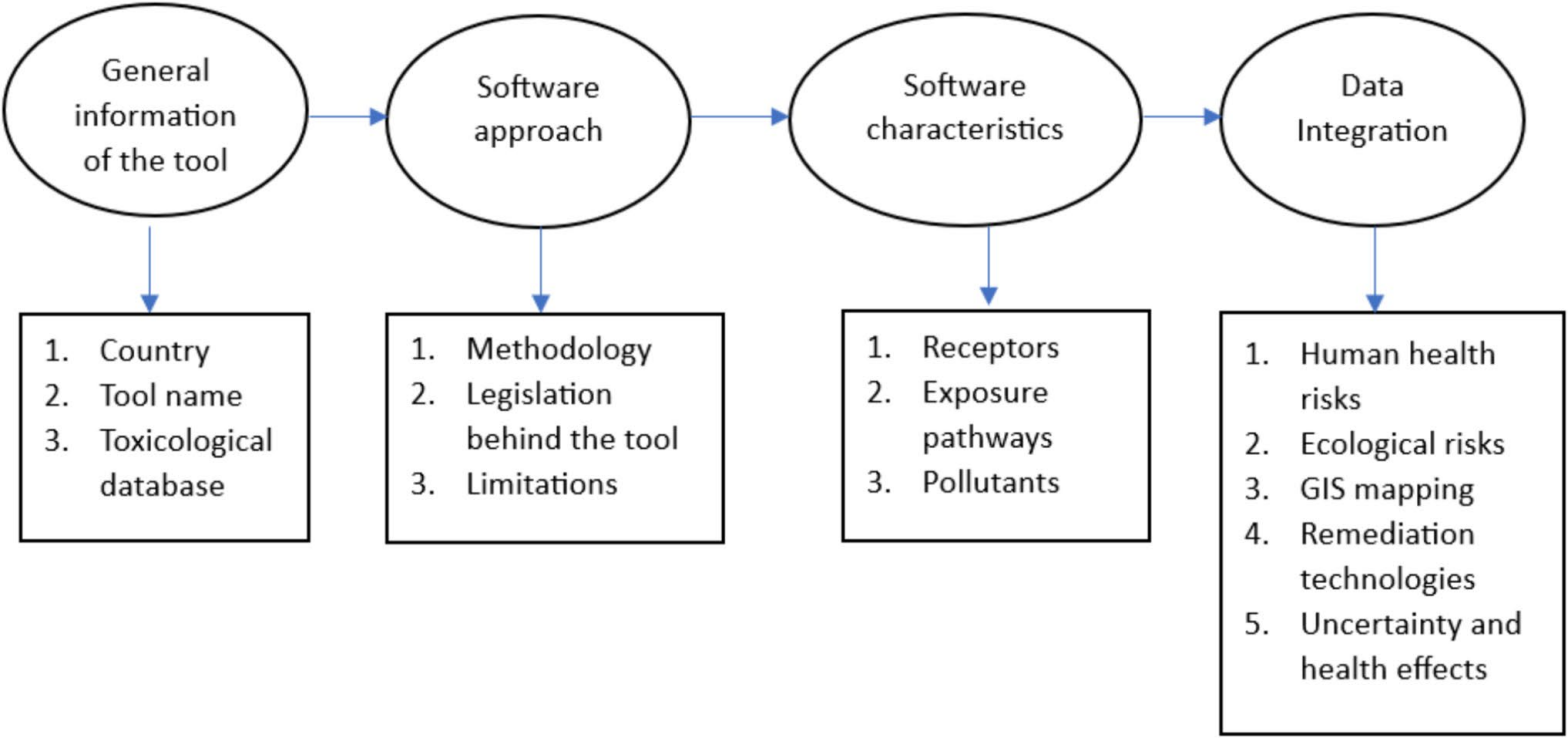
The main points used to analyze the software tools identified, adapted after Mahammedi et al. (2020)

Moreover, the information presented on these tools is based on the available range of scientific research and datasets. The comparison was made considering the available technical guidance for each tool and articles. The review paper did not consider the software tools which lack available online information in English on the subjects discussed. In this framework, 12 approaches used across Europe were evaluated, encompassing the legislative context leading to the development of the tools (Payá Pérez and Rodríguez Eugenio 2018), conceptual model, and other functions. The identified tools were analyzed based on the principles set out in Fig. 3.

#### Regulation in force behind the implemented software tools

There are some review papers carried out on the available HHRA tools. For example, Swartjes (2015) approached the theme of biomonitoring, with a bigger attention toward exposure model comparison. Consequently, Loos et al. (2010) have approached a comparison of human and wildlife receptor-oriented exposure models. Afterward, Hoek and colleagues (2018) conducted a review on monitoring and modeling of human health exposure on the seven tools used in EU countries, while Mahammedi et al. (2020) have appraised the existing HHRA tools in terms of complexity level ranging from preliminary (PRA) to generic quantitative (GQRS) then detailed quantitative risk assessment (DQRS). Moreover, Zhang et al. (2023) have extended the knowledge on the HHRA steps, methods, models available and research programs conducted to date on HHRA.

Even though many tools were developed worldwide, the current review paper is focused on identifying the legislative context, receptors and pathways, pollutants considered, and integrated data in the 12 tools (Table 1). The subject tools considered in the review were CLEA (UK), CSOIL (Netherlands), JAGG (Denmark), RBCA Toolkit (US), Risk-net (Italy), CalTOX (US), S-RISC (Belgium), RISC5 or BP RISC (US), RECOLAND/REMPET (Romania), CARO-PLUS (Germany), Rome plus (Italy), and K-RBCA (South Korea). As both tools RECOLAND and REMPET present mostly the same characteristics, except for REMPET which included an additional feature, we will review both tools as one integrated model approach.

The growing public concern about the harmful effects on the environment and human health generated by soil pollution has generated legislative actions on the pollutants released into the environment. Regulation on soil contamination and remediation has emerged in the USA through the “Comprehensive Environmental Response, Compensation and Liability Act in 1980” (Jennings 2008), known as “CERCLA” or “Superfund,” after the Stockholm Declaration on the Human Environment in 1972 (Azam 2016). The Act is envisioned to address the pollution of uncontrolled or abandoned polluted sites. The amendments were made in 1986 via the “Superfund Amendments and Reauthorization Act (SARA)” (US EPA 1980). In the same way, the Resource Conservation and Recovery Act (RCRA) governs the treatment, storage, and disposal of hazardous waste, including petroleum contamination (Gerrard and Lester 2006). Many US states have established their own standards for clean-up (or remediation) levels in the lack of a harmonized legislative concept at the national scale (Jennings 2008; Lombi et al. 1998). As such, there may be differences in the levels of contamination cleanup. Meanwhile, the modeling tool, as such, is the case of RBCA Toolkit, CalTOX, and RISC5 or BP RISC that were developed to support the process of risk evaluation of soil and groundwater.

The situation in Europe is comparable: individual countries have adopted distinct clean-up values in the absence of coordinated EU-wide legislation specifically on soil (Lombi et al. 1998; Rodrigues et al. 2009), although necessary actions are ongoing.

Netherlands was among the first countries worldwide to adopt an environmental policy called “Interim Soil Remediation Act” in 1983 (Swartjes et al. 2012), followed by the “Soil Protection Act” in 1987, which was amended multiple times. The last revision of the Dutch Soil Protection Act added the supplement “Ministerial Circular on Soil Remediation”, which came into force in 2013. The legislation has the aim of addressing the existing soil contamination and preventing new contamination cases (Swartjes et al. 2012; Boekhold 2008; Römbke et al. 2005). Based on the legislation, the concentrations of contaminants in soil and groundwater are analyzed against soil quality standards (SQS) in the context of clean-up levels, which are divided into two: “background values” and “intervention values.” Given the SQS results, the site can be categorized either as “clean soil,” “slightly contaminated soil,” or “seriously contaminated soil.” In the context of clean-up levels, the Dutch legislation “background” and “intervention” values are dependent on soil type (Swartjes et al. 2012).

In Romania, the first regulation on environmental pollution was issued as “Ministerial Ordinance 756 from 1997”. The regulation presents the National contaminants concentration characterization based on “normal values,” “alert threshold values,” and “intervention threshold values” for sensitive (agricultural means) and less sensitive (industrial) site use (Environmental Ministry of Romania 1997). The values have been set up for the clean-up or remediation options. In the case of a contamination resulting in “alert threshold values” categorization, further actions are dependent on the Environmental Protection Agency’s guidance. On the other hand, the “intervention threshold values” require the decision of the local authority regarding the development of the risk analysis specific to the location and impose immediate actions toward the rehabilitation of the initial soil state (Parliament of Romania 2019; Environmental Ministry of Romania 2020).

In Denmark, the first regulation considering the contamination of the soils came into force in 1983; later, in 1990, it was revised as the “Waste Landfill Regulation” (*Affaldsdepotloven*) (Rosen 2010). Later on, Denmark promulgated the “Act on Soil Contamination” in 1999 (Ministry of Environment and Energy of Denmark 1999), which was last updated in 2009 (Ministry of Environment and Energy of Denmark 2009).

Similarly, South Korea has adopted its first legislation on preventing potential hazards resulting from soil contamination in 1995, last updated in 2024, via “Soil Environment Conservation Act” (South Korea Government 1995).

At the same time in Italy, the first regulation oriented toward environmental protection was “Ministerial Decree 471/1999”. Later was revised and reinstated as “Legislative Decree 152/2006” and its amendments (Ministry of Environment and Energy Security 2006) set out an applicable legislative framework dedicated to environmental protection, including soil, water, and air. Complementary documents in relation to soil and water pollution were developed, such as National Standards dedicated to contaminated sites assessment by the Agency of Environmental Protection and Technical Services (APAT) and risk analysis application specific to the location, MATTM (Ministry of the Environment and Protection of Land and Sea 2014, D’Aprile et al. 2008), and the HHRA tool use (ISPRA 2019). In the context of Italian legislation on contaminated sites, it is primarily aligned with the ASTM E-1739 and ASTM PS-104 Standards, using a database issued by ISS and INAIL (ISPRA 2019).

In this context, the designed tools are conceived to satisfy the needs in the risk analysis study based on the legislative requirements and provide rapid results (Table 1). The database (physicochemical and toxicological properties) used by the models is adapted based on the National policies and legislation. For instance, the CLEA model uses the latest information published within the reports of Toxicological (TOX) and Soil Guideline Values (SGVs) of the Environmental Agency in the UK (Jeffries and Martin 2009). Risk-net operates based on the ISS-INAIL database as described in the Italian Institute for Environmental Protection and Research (ISPRA) Methodologies (ISPRA 2024). Recoland/Rempet utilizes the Environmental Protection Agency (US EPA) database (US EPA 2024b), while the RBCA toolkit offers the possibility of multiple customized database files and also proposes default physical, chemical, and toxicological parameters published by the Texas Commission on Environmental Quality and from published sources in the USA, Netherlands, and the UK, subject to regular updates (GSI Environmental 2023; Chang et al. 2004). RISC5 or BP RISC uses the IRIS database (Spence and Walden 2013). The JAGG database for PFAS compounds is taken from the Regional Knowledge Center for Environmental Resources (VMR) revised handbook (Danish Environmental Protection Agency 2022). As for K-RBCA, the US EPA toxicological and physicochemical data has been adopted, as most appropriate with the legislative context (Nam et al. 2011a, b).

The *CLEA* model estimates the exposure of children and adults to soil contaminants based on the conceptual model for site conditions (Conceptual Site Model, CSM) and human behavior over a timeframe, and general assumptions of the fate and transport of chemicals in the environment (Jeffries and Martin 2009).

The *CSOIL* model was developed in line with the previous models such as SOILRISK, RIVM model, and HESP, which calculates the risk related to human behavior and soil contamination, using the fugacity theory and partition coefficients of the contaminants’ distribution in soil and the human exposure (Brand et al. 2006).

The *JAGG* program is used to perform risk assessment of contaminated sites, incorporating the fugacity calculations, evaporation indoor and outdoor, migration of the contaminant to the unsaturated zone, convective migration of gas from landfills, diffused soil contamination, groundwater contamination, probability calculation, and transport through low-permeability fractured media (Danish Environmental Protection Agency 2000; Chambon et al. 2011).

The *RBCA Toolkit* risk assessment modeling tool was developed considering the ASTM standard for Risk Based Corrective Action, has incorporated a comprehensive fate and transport model for air, groundwater and soil exposure pathways, with the possibility of use for site-specific environmental clean-up risk evaluation (GSI Environmental 2023).

The *Risk-net* tool allows us to evaluate the human health risk for different exposure pathways through fate and transport models aligned with the ASTM standard and ISPRA guidelines. The model allows calculation of the risk and remediation targets, such as site-specific target levels (SSTL).

The *S-RISK* tool is a human exposure to contaminated sites model enabling the calculation of screening levels for generic and site-specific lay-out and derives associated human health risks. The tool represents the updated version of RISK-HUMAN in Belgium. It is a steady-state mass conservation model for estimating human exposure and risk to pollutants in soil and groundwater and incorporates both inorganic and organic pollutants (Goidts et al. 2018; Cornelis et al. 2022).

The *RISC5* is a risk assessment tool which calculates soil and groundwater clean-up targets and has incorporated several models, e.g., the Johnson and Ettinger model, plant uptake, vapor intrusion, surface water mixing, and ecological food web model (Spence and Walden 2013).

The *CalTOX* is a multimedia fugacity fate, transport, and exposure model designed for site-specific risk assessment approaches (Fei et al. 2012; Coulibaly and Labib 2001; Moklyachuk et al. 2014). The CalTOX tool is known for the multimedia transport and transformation model, using a variety of scenario exposure pathways and as an add-in to quantify and evaluate the uncertainty and variability, and sensitivity analysis (Suciu et al. 2012; Uribe-Hernandez et al. 2010).

The *RECOLAND/REMPET* tools are US EPA-based exposure models for predicting risks associated with contaminated soil, supporting management of contaminated sites implying legislative context and remedial options. The tools have incorporated a plant and animal uptake model (Istrate et al. 2016).

The *CARO-PLUS* is a decision support tool–system dynamics model dedicated to preliminary assessments as part of a tiered approach, allowing us to simulate, provide a cost-efficient analysis of a possible remediation option, and understand the effects of remedial actions, identify the contaminant source, analyze the pollutant behavior in different media, and manage groundwater plumes contamination. CARO-PLUS addresses the (a) contaminant mass-release from organic mixtures, (b) solute transport in groundwater, (c) human health risks, and (d) uncertainty of the assessment. It is divided into three modules: source release, contaminant transport, and human health assessment (Mcknight et al. 2010; McKnight and Finkel 2009). The source release model is presented using Raoult’s law (McKnight and Finkel 2013).

The *ROME plus* is a risk assessment modeling tool and includes two suites: “San Giovanni”—applicable for risk analysis considering soil–gas measurements and “Fori”—flux chambers, as per the SNPA Guidelines (ISPRA 2019).

The *K-RBCA* is a tool developed in line with the Korean soil legislation to perform human health risk assessments using equations on determining risks derived from soil and groundwater considering US EPA and ASTM guidelines. The tool is similar to the commercial RBCA Toolkit Chemical Release, having a distinct “operable unit, OU” function characteristic to the Korean legislation, which allows for adding different contaminant concentrations at the site level assessment (Nam et al. 2011a, b).

### HHRA tools: exposure–receptor

In this context, the term carcinogenic risk (CR) represents the incremental probability of developing cancer due to exposure to one or more mutagenic carcinogenic compounds (non-threshold contaminants). Among different countries, the acceptable lifetime CR ranges from 10^−6^ to 10^−4^, which corresponds, respectively, to the probability of 1 in 10,000 or 1 in 1,000,000 individuals developing the disease (Carlon 2007). The different tools have incorporated cancer risk based on the chosen model and legislative context of the origin country, e.g., Risk-net assumes a CR of 10^−6^, and ROME PLUS assumes a CR of 10^−6^ (SNPA and ISPRA 2021); similarly, a CR target was considered in CalTOX (Coulibaly and Labib 2001), RECOLAND/REMPET, and K-RBCA at 10^−6^ (Nam et al. 2011a, b) and S-RISK at 10^−5^ (Cornelis et al. 2022); the RBCA Toolkit allows specifying the lifetime risk limits between the given range (Connor et al. 2007) or CSOIL, which uses 10^−4^ under the intervention values based on a relevant exposure scenario and 10^−6^ for maximal values considering land use specific exposure scenarios (Swartjes et al. 2012). The non-carcinogenic risk is quantitatively expressed as hazard index (HI) or risk index (RI) that is calculated as the ratio between the contaminant dose to which a receptor is exposed and the corresponding reference dose, RfD (reference dose, mg/kg day) represents an estimation of average daily exposure which has no expected adverse effects on human health during the lifetime, (non-threshold contaminants). Therefore, the HI value typically considered acceptable is 1 (National Environment Protection Council (NEPC) Australia 2011; D’Aprile et al. 2008).

**Fig. 4:**
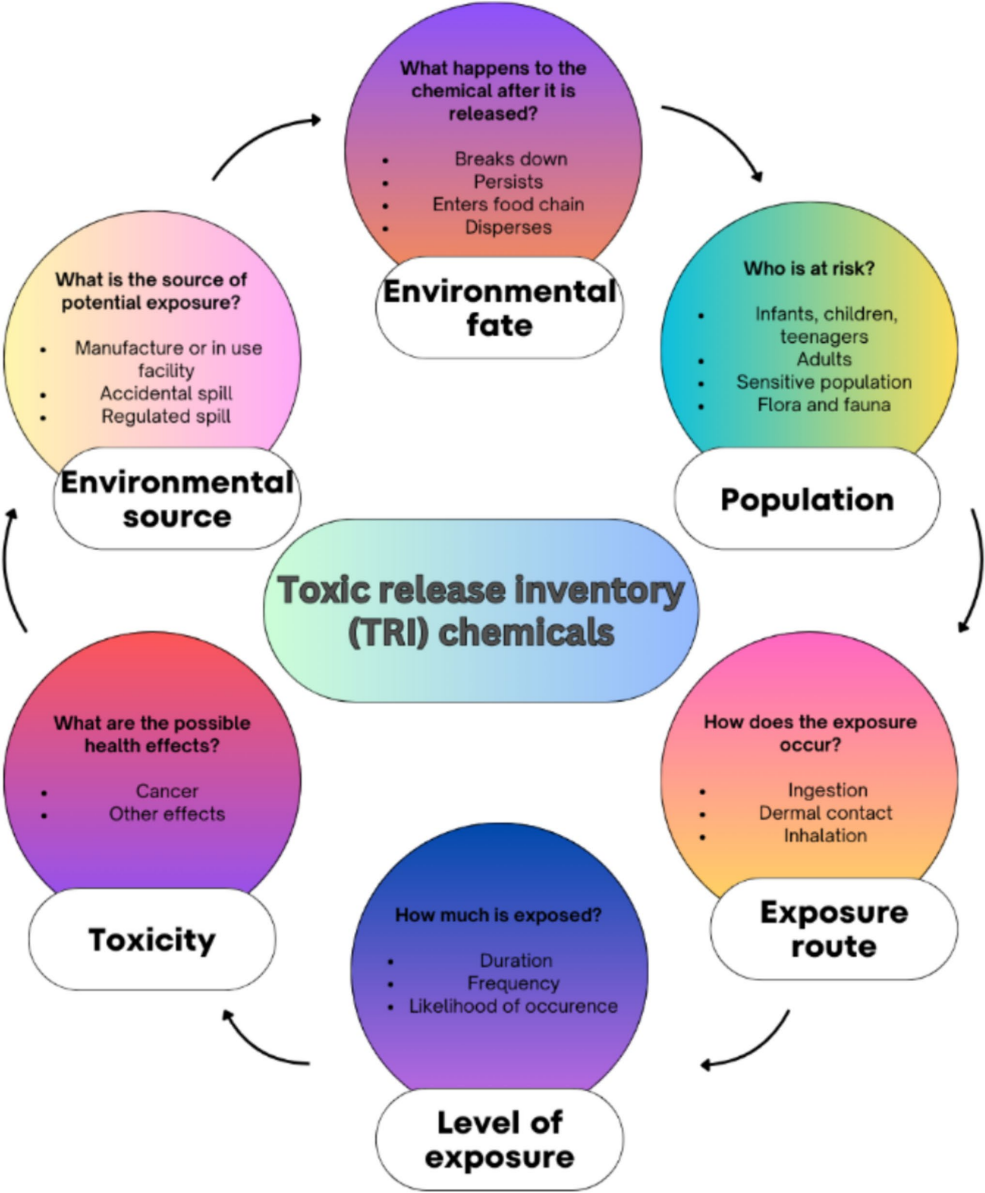
Factors influencing the risk, adapted after US EPA potential risks from TRI chemicals

In general, the *source–exposure pathways–receptor* concept has been implemented following the framework set up by the National Research Agencies and legislation from each country. Exposure refers to the interaction, overtime and space, between an individual and one or more biological, chemical, or physical agents through soil, dust, air, water, or food. This concept is widely used by scientists to evaluate how people come into contact with hazardous substances, considering the factors (Fig. 4) leading to potential human health risks (MacIntosh and Spengler 2000; FAO and UNEP 2021b; USEPA 2024).

As described in Geraets et al. (2016), the prevailing step in the risk assessment is considered the determination of exposure concentration, dose, exposure frequency, and exposure duration. It is crucial to understand the exposure factors by performing surveys and measurements to determine site-specific estimates, such as duration (short-term, long-term), frequency, and magnitude (intermittent or peak exposures), and other general exposure factors which contribute to transparent rather than conservative results (MacIntosh and Spengler 2000; Yoon et al. 2020; Barrio-Parra et al. 2019).

#### Exposure frequency and duration

Exposure frequency (EF) is represented by the frequency at which the individuals are exposed to contaminants and is determined in days per year (d y^−1^) (US EPA 1992; Miletic et al. 2023). The default values for EF are different in each tool depending on the policy frameworks. For instance, CLEA utilizes an EF of 365 d y^−1^ for children, adults, and seniors between 1 and 75 years and 180 d y^−1^ for children between 0 and 1 year (Sun et al. 2020). S-risk uses the same default EF of 365 d y^−1^ for all scenarios (Cornelis et al. 2022), while Risk-net, ROME plus (SNPA and ISPRA 2021), RECOLAND/REMPET (Cocârță and Stan 2017), and RISC4 or BP RISC (Spence and Walden 2001) consider 350 d y^−1^ for children and adults under the residential/recreational/agricultural scenario and 250 d y^−1^ for industrial receptors. EF values in the RBCA Toolkit match with US EPA guidelines, implementing default values of 350 d y^−1^ for residential land use and 250 d y^−1^ for commercial/industrial and most likely exposure (MLE) of 40 d y^−1^ in both cases (GSI Environmental 2023). The same input EF parameters were considered in K-RBCA (Nam et al. 2011a, b).

Exposure duration (ED) is represented by the timeframe at which the individual is exposed to the contaminants of concern (USEPA 2014). The default ED value in CLEA software is 1 year for individuals between 0 and 16 years of age, 49 years for individuals between 16 and 65 years of age, and 10 years for individuals between 65 and 75 years (Jeffries and Martin 2009). The default ED values in Risk-net are 6 years for children, 10 years for teenagers, 24 years for adults, 5 years for seniors, and 25 years for workers. RISC4 or BP RISC has an ED of 5 years for children and 30 years for adults, considering a residential scenario (Spence and Walden 2001). CalTOX considers the ED based on the exposure scenario selected by the user (Åberg et al. 2015). As for the K-RBCA, the ED under the residential scenario is 30 years, and 25 years for an industrial scenario (Nam et al. 2011a, b).

#### Exposure pathways (EP)

**Fig. 5:**
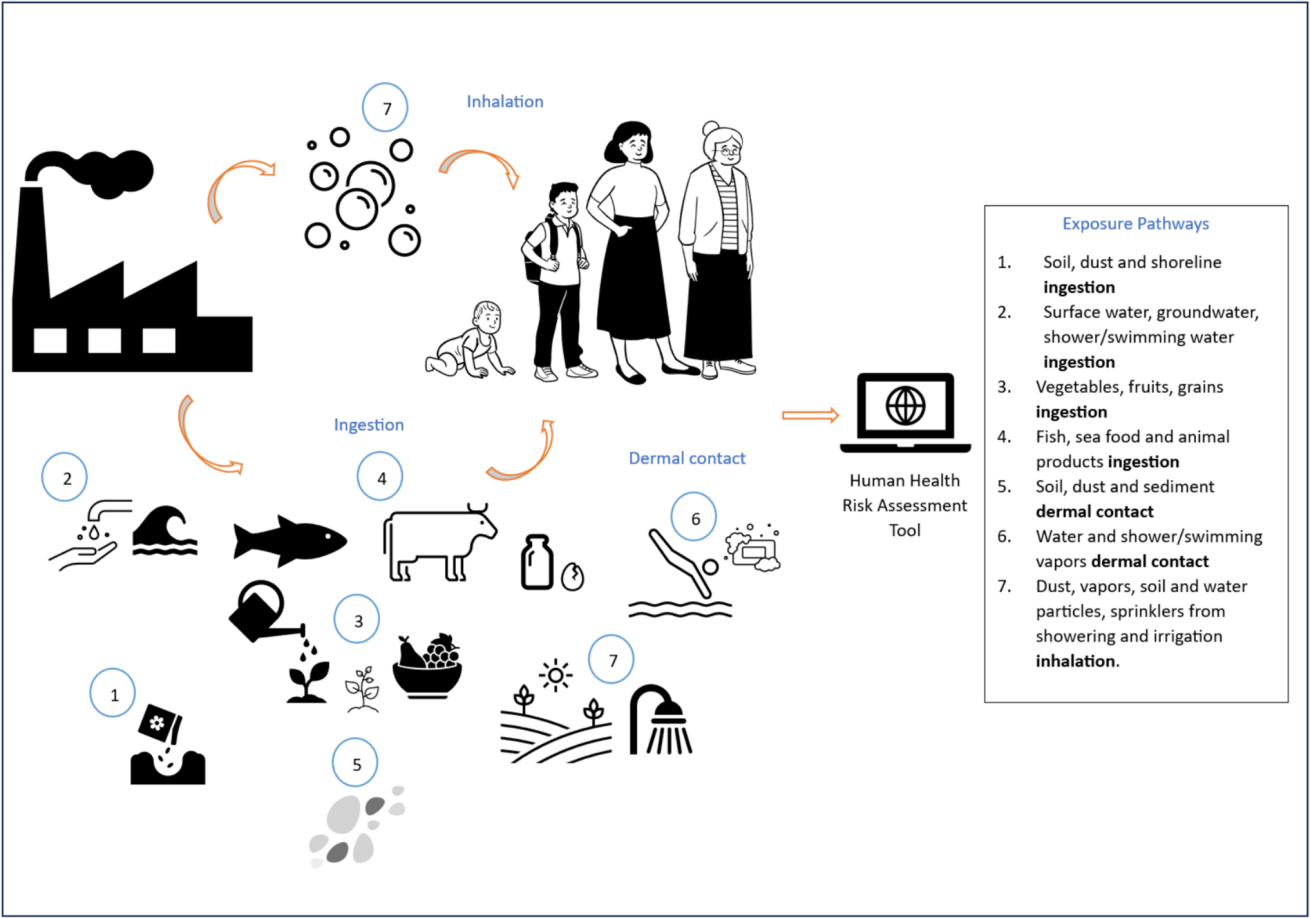
Basic scheme of source–exposure pathway–receptor route based on CSM and computing risk data presentation

The several models examined enable the consideration of distinct exposure pathways (EP) in the context of the assessment of risks related to the presence of pollutants in environmental matrices. Figure 5 illustrates the pathways that are taken into account in the context of HHRA based on the analyzed references on the tools’ usability (Pinedo et al. 2014).

Table 2 reports the specific exposure pathways (EP) considered in the analyzed tool. It can be noticed that not all the EPs shown in Fig. 5 were covered integrally in all the tools.

**Table 2:** Exposure scenarios considered in the evaluated models (Mcknight et al. 2015; McKnight and Finkel 2013; Nam et al. 2011a, b; SNPA and ISPRA 2021; DEFRA 2015; Breemen et al. 2020; GSI Environmental 2023; Sun et al. 2020; Cornelis et al. 2022; Spence and Walden 2013; McKone TE and Lawrence Livermore National Lab CA 1993; SNPA and ISPRA 2021)

Tools	Receptors				Scenario*	Exposure pathway**			*Outdoor*	*Indoor*
	*Adults*	*Children*	*Workers*	*On-site/off-site*		*Oral*	*Dermal*	*Inhalation*		
(1) CLEA			-	*On-site*	c, d, e	Direct soil, dust, grown vegetables, soil attached to vegetables	Soil and dust	Dust and vapors from soil		
(2) CSOIL			Adults	On-site	a, b, c, d, e, f	Soil particles, local vegetables, soil contaminants via drinking water from pipelines	Soil and water during showering and bathing	Soil particles, vapors from soil, vapors during showering		
(3) JAGG	-	-	-	On-site	-	Low permeability fractured media (fugacity, vertical transport, indoor and outdoor air and groundwater) risk assessment				
(4) RBCA Toolkit				On-site and off-site	b, c, d, e	Soil, water (drinking, swimming), vegetables and fish consumption	Soil and water	Dust (outdoor only) and vapors		
(5) Risk-net				On-site and off-site	a, b, c, d	Soil, direct groundwater or from leaching to groundwater, vegetable	Soil	Particulate and vapors		
(6) S-RISK				On-site	a, b, c, d, f	Soil, indoor settled dust, vegetables, animal products (meat, milk, eggs), drinking water and groundwater	Soil and dust, water during bathing and showering	Vapors, particles from the air, vapors during showering		
(7) RISC5 or BP RISC				On-site	b, c, d	Soil and sediment, drinking water/surface or irrigation, home-grown vegetables (grown in contaminated soil or watered)	Soil and dust, water while showering or swimming	Dust (*outdoor only*) and vapors (*indoor only*), sprinkles (showering, irrigation)		
(8) CalTOX	-	-	-	On-site	a, b, d, e	- Fruits, vegetables, grains via air transfer to plants - Meat, milk, eggs via transfer from air to plants afterward to animals - Meat, milk, eggs consumed via animal inhalation exposure - Mother’s milk exposed to soil, water, air contaminants - Soil, transfer contaminant via soil - Tap water, irrigated fruits and vegs, contaminant transfer via water, fish and sea food, swimming or other type of water recreation	Soil, baths and shower vapors, and swimming	Soil vapors and particles; shower vapors, air gases and particles		
(9) RECOLAND and REMPET*				On-site	a, b, d	Soil, meat (beef, chicken, pork), eggs, dairy, vegetables (leafy, root, protected and exposed)	Soil	No		
(10) CARO-PLUS					a, b	Drinking water, leafy vegetable, soil, fin fish, shoreline sediment, shower and swimming water	Soil, swimming, shower, sediment	Re-suspended soil, air, indoor shower		
(11) ROME Plus				On-site and off-site	b, c, d, e	No	No	Soil–gas and flux chamber		
(12) K-RBCA				On-site	-	Soil and groundwater	Soil	Dust and vapors		

Hyphens (-) denote “no information available when using online search engines”

^*^Scenario: a, agricultural; b, industrial/construction; c, recreational; d, residential; e, commercial; f, nature/parks

For instance, in the case of CLEA tool, before its implementation, the UK government performed a survey on a number of households, between the period of 2003 and 2005, acquiring housing information. CSM has been developed in accordance with the results obtained during the surveys (Jeffries and Martin 2009).

##### Oral EP

The Ingestion EP in the context of the models developed (Table 2) covers:

- Soil (soil, dust, shoreline sediment)
- Water (groundwater and/or pipeline water, swimming, shower)
- Food (vegetables, seafood, animal products, mother’s milk)

The analyzed model tools are covering the oral exposure through different pathways; the most common is soil ingestion and vegetable consumption exposed to contaminated soil (Swartjes et al. 2012; Lee et al. 2018; Du et al. 2022). On the other side, a few tools (namely S-Risk, CalTOX, RECOLAND/REMPET) have considered the animal product ingestion (meat, eggs, milk), which have been exposed to contaminated soil (Cornelis et al. 2022; McKone 1993a; Bierkens et al. 2011), except for the RBCA toolkit and CARO-PLUS, which consider fish ingestion (Petruta and Drăcea 2023). Moreover, CalTOX has implemented the ingestion of mother’s milk (McKone 1993a).

Groundwater drinking has not been considered in all the tools. Some models assess direct drinking from groundwater wells, while in others the household water drinking from municipal tap water is applied. For example, direct consumption of groundwater cannot be assessed in CSOIL as it is assumed that the population is drinking water that underwent treatment quality. However, the CSOIL model considers the option of plastic pipelines drinking water that may be located in a contaminated zone, where the contaminant can enter via permeation in the drinking water stream (Breemen et al. 2020; Swartjes et al. 2012). A similar option is implemented in the S-Risk model (Cornelis et al. 2022). Direct drinking from groundwater wells is being evaluated in some tools, for instance, in Risk-net, CARO-PLUS (McKnight and Finkel 2013), and others.

Beside the calculation of the intake of direct groundwater drinking RISC5, CalTOX has the option of ingestion of irrigated or surface water, which may occur mostly to children while playing outdoors, or when contaminated water is used to fill the swimming pools (Spence and Walden 2013; Coulibaly and Labib 2001).

On the other hand, CLEA and RECOLAND/REMPET models do not have the option to calculate the risks related to the drinking water pathway from on-site source or water plastic–pipeline permeation (Jeffries and Martin 2009).

##### Dermal EP

Dermal exposure to contaminants may occur from direct (dermal contact with contaminated soils or water) or indirect contact (contact while cleaning indoor accumulated contaminated dust)(US EPA 1992).

Similarly, in the case of Oral EP, the dermal contact route can be assessed in all the tools evaluated and permits obtaining HHRA data. The software tools provide risk output information on dermal EP; however, some of them consider different contaminated media, as follows:

- *Soil or dust* (gardening, construction activities, cleaning, etc.): assessment is available in all the discussed tools, except for ROME plus (SNPA and ISPRA 2021) and JAGG (Danish Environmental Protection Agency 2000).
- *Water* (during bathing or swimming, etc.): option is available in CSOIL (Breemen et al. 2020), RBCA Toolkit (GSI Environmental 2023), S-Risk (Cornelis et al. 2022), CalTOX (McKone 1993b), RISC5 (Spence and Walden 2013), and CARO-PLUS (McKnight and Finkel 2013).
- *Sediments* (fish, shoreline): option is available only in CARO-PLUS (Mcknight et al. 2010).

##### Inhalation EP

The EP via inhalation of the contaminant can be derived from aerosols (fine particulate matter such as sprays), particulate matter (e.g., dust), or vapors (volatile and semi-volatile pollutants) (US EPA 2021b). In the case of a polluted site, the contaminant can migrate from surface or subsurface soil and groundwater to the air (vapors) or be transported from surface soil by the wind (dust) into the atmosphere. Once in the atmosphere, these can be inhaled by the receptors living on the site or in the immediate vicinity (US EPA 1997, 2021b).

Inhalation of contaminants can result from:

- *Surface soil/topsoil* (particles, dust and/or volatilization of contaminants): S-RISK (Cornelis et al. 2022), Risk-net, RBCA Toolkit (Connor et al. 2001), CalTOX (McKone 1993a), K-RBCA (Nam et al. 2011a, b), RISC5 or BP RISC (Spence and Walden 2013), CLEA (Jeffries and Martin 2009), CARO-PLUS (McKnight and Finkel 2013), and CSOIL (Breemen et al. 2020).
- *Subsurface soil* (volatilization of the contaminants): S-RISK (Cornelis et al. 2022), Risk-net, RBCA Toolkit (Connor et al. 2001), CalTOX (McKone 1993a), K-RBCA (Nam et al. 2011a, b), RISC5 or BP RISC (Spence and Walden 2013), CLEA (Jeffries and Martin 2009), and CSOIL (Breemen et al. 2020).
- *Groundwater* (volatilization of contaminants): S-RISK (Cornelis et al. 2022), Risk-net, RBCA Toolkit (Connor et al. 2001), and CalTOX (McKone 1993a).
- *Surface water* (sprinklers/vapors from showering and/or irrigation): CSOIL, RISC5, and CalTOX for both indoor and outdoor scenarios (Spence and Walden 2013; Breemen et al. 2020; McKone 1993a). CARO-PLUS has incorporated indoor shower inhalation (McKnight and Finkel 2013).

Determination on risk derived from contaminant inhalation in the current tools is evaluated based on the technical references nationally recognized and adopted by each model. For example, RBCA toolkit (GSI Environmental 2023), K-RBCA (Nam et al. 2011a, b), Risk-net, Risc5 or BP RISC (Spence and Walden 2013), CLEA (Jeffries and Martin 2009), CARO-PLUS (McKnight and Finkel 2013) tools developed following ASTM-RBCA tiered analytical approach procedure (ASTM 2022), quantitatively determine CR and non-CR to human health via inhalation through the application of fate and transport (FT) model of the contaminant in soil and groundwater (Mangiapia et al. 2023). The FT model adopted in S-Risk follows US-EPA 1996b (topsoil contaminant volatilization) and RBCA (subsurface soil layers and groundwater volatilization) procedures equation (Cornelis et al. 2022). CSOIL has implemented the FT model (particles and volatile contaminants) for the inhalation EP based on the RIVM Reports (Van Den Berg 1995; Waitz et al. 1996). Rome plus has adopted the FT model indoor and outdoor, on-site and off-site based on the Italian guidelines (SNPA and ISPRA 2021), while CalTOX has fully aligned with the US EPA and DTSC guidelines (McKone 1993a).

Ultimately, the models have adopted certain EP (as presented in Table 2) limited to the domestic regulation.

Complementary to the above mentioned, ROME plus and Risk-net allow usage of flux-chambers and soil-gas data for the assessment of inhalation risks (SNPA and ISPRA 2021).

#### Receptors

Previous research articles highlighted differences between the models from the type of exposure pathways perspective to the required input data for the receptor parameters (Lee et al. 2018; Sun et al. 2020). Age groups play a pivotal role in the HHRA model, due to different risks arising while in contact with the contaminant. For instance, children are vulnerable to contaminants due to their growing body and activities (e.g., hand-to-mouth) potentially leading to higher risks compared to adult receptors. On the other side, elderly people have a lower immune system, which raises the risk potential (Goel 2006; Cheng and Nathanail 2009; Dixit and Roy 2016). Different studies have emphasized the age-related vulnerabilities which influence health outcomes (Pirastu et al. 2013; Alam et al. 2021; Amarillo et al. 2014). Given these insights, the model developers incorporated age-specific input data tailored to the legislative context of the country where it is developed. Considering the sensitivity of the age categories, most of the models allow user-defined input values, thus enabling the user to modify the information applicable to the assessment. Based on the evaluated tools, the general approach of the receptor age category is defined in Table 3.

**Table 3:** Human being receptor category adopted in the models evaluated (Suciu et al. 2012; Jeffries and Martin 2009; Brand et al. 2006; Nam et al. 2011a, b; Danish Environmental Protection Agency 2000; GSI Environmental 2023; Cornelis et al. 2022; Spence and Walden 2013; McKone 1993b; SNPA and ISPRA 2021)

Receptor	Child	Adult
CLEA	0–16 y (staggered data)	16–65 y, 65–75 y
CSOIL	0–6 y	6–70 y
Jagg	1–3 y	20–60 y
RBCA toolkit	6 y, 12 y	30 y
Risk-net	0–6 y, 7–16 y	16–65 y, > 65 y
S-Risk	1–3 y, 3–6 y, 6–10 y, 10–15 y, 15–21 y	21–61 y, > 61 y
Risc5	6 y	24 y
CalTOX	-	-
RECOLAND/REMPET	0–6 y	70 y
Caro-plus	x	x
Rome plus	0–6 y, 7–16 y	16–65 y, > 65 y
K-RBCA	x	x

(-) Not considered, user defined input parameters required.

(x) No information available in the tool presentation.

##### Environmental factors in assessing receptors’ risk

The tools have incorporated (at the input section) a set of default environmental properties from diverse databases such as meteorological, hydrological data, soil properties needed to run the models for human risks evaluation. The environmental parameters are used particularly in the tools which have adopted fate and transport models. Among the listed tools, some of them allow user-defined adjusted parameters. For instance, the models account for soil pH level, except for the RECOLAND/REMPET tool which did not develop a fate and transport model and does not require environmental parameters. Moreover, some tools such as Risk-net offer the possibility to adjust the soil pH based on the obtained field data, while other tools, as is the case of RISC5, use a predefined pH level (pH, 7) (Spence and Walden 2013; Chang et al. 2004). For example, the soil pH is responsible for the soil–water partition coefficient (*k*_d_) of certain pollutants found in soil (e.g., metals), which further can contribute to the increase or decrease of the overall exposure risk (Zeng et al. 2011). Among other environmental variables tackled within the tools are organic matters, such as organic-carbon content, except for the RECOLAND/REMPET; ambient environmental temperature included in CalTOX, RBCA, CLEA, CSOIL, and S-Risk (DEFRA 2015; Breemen et al. 2020; GSI Environmental 2023; Sun et al. 2020; Cornelis et al. 2022; Spence and Walden 2013).

Environmental parameters are important in the determination of risk results when considering a wide array of exposure pathways involving different types of receptors, thus avoiding the overestimation and yield of conservative results (Li 2020; Li and Niu 2021). Sensitive receptors, especially children, are included in the tools as a separate category and are differentiated from the adult receptors by their body weight, soil and water ingestion rate, soil ingestion fraction (Risk-net, RECOLAND/REMPET), skin surface area, and exposure duration. Moreover, Risk-net and Rome plus have included an additional factor, namely the age-dependent adjustment factor (ADAF), herein accounting for children’s higher susceptibility to exposure to certain carcinogenic and mutagenic pollutants in early life exposure (SNPA and ISPRA 2021; Li 2018).

### HHRA tools by pollutants

The models considered have incorporated a default chemical database which includes physicochemical and toxicological parameters of different contaminants typical of concern at contaminated sites. Contaminant information in most cases is visible to the user, such as RBCA toolkit, Risk-net, or S-RISK. Some tools also have the possibility, besides the visualization, to export and import user-defined databases, as is the case of Risk-net. The models evaluated consider diverse classes of chemicals (Table 4), but metals, BTEX, and PAHs are covered in mostly all the tools. While the models discussed in the current review have incorporated both carcinogenic and non-carcinogenic contaminants which exhibit toxic properties, RECOLAND/REMPET were focused on the carcinogenic chemicals only, part of Group 1 IARC classification (carcinogenic to humans) (WHO 2024).

**Table 4:** Contaminants considered in the assessed models (DEFRA 2015; Otte et al. 2001; Danish Environmental Protection Agency 2016; GSI Environmental 2023; Spence and Walden 2001; McKnight and Finkel 2013; SNPA and ISPRA 2021; Nam et al. 2011a, b; Yang 2012; Msibi et al. 2023)

Tool	Class of contaminants	Number of chemicals in the database
CLEA	Metals, BTEX, PCBs, dioxins and dioxin-like compounds and phenols	30 chemicals
CSOIL	Metals, BTEX, PAHs, Chlorinated hydrocarbons, dioxins and dioxin-like compounds, PCBs, pesticides, mineral oil, phthalates, cyanides, per- and polyfluoroalkyl substances (PFOS and PFOA), and other compounds	Not specified
JAGG	BTEX, PAHs, aromatic and aliphatic hydrocarbons, hydrocarbon mixtures, PFAS, and other compounds	192 chemicals, 26 PFAS compounds
RBCA toolkit	Metals, BTEX, PAHs, chlorinated hydrocarbons, halogenated aliphatics, nitrobenzenes, chlorobenzenes, chlorinated and non-chlorinated phenols, ammines, pesticides, dioxin, PCBs, hydrocarbons, and other substances	650 chemicals
Risk-net	Metals, BTEX, PAHs, chlorinated hydrocarbons, halogenated aliphatics, nitrobenzenes, chlorobenzenes, chlorinated and non-chlorinated phenols, ammines, pesticides, dioxins, PCBs, hydrocarbons, and other substances	144 chemicals
S-Risk	Not specified	128 chemicals
RISC5	Metals, BTEX, PAHs, phthalates, dioxin, aromatic and aliphatic hydrocarbons, and other substances	Not specified
CalTOX	PAHs, Hexachlorocyclohexanes (HCH), phthalates, pesticides, TPH, phenols and other substances	Not specified
RECOLAND/REMPET*	Metals, PAHs, BTEX (*REMPET only*), PCBs (*RECOLAND only*)—carcinogenic compounds only	32 chemicals
CARO-PLUS	LNAPLs and DNAPLs	Not specified
ROME plus	BTEX, PAHs, hydrocarbons, phenols and chlorinated phenols, chlorobenzenes, nitrobenzenes, aliphatic halogenated compounds, and other substances	70 chemicals
K-RBCA	Metals, BTEX, PAHs, total petroleum hydrocarbons (TPH), BTEX, PAHs, aliphatic hydrocarbons	Not specified

The difference in the amount and type of chemicals included in the tools denotes the diverse scope of tool development, and the discrepancy given by the regulation in force in each country and methodologies applied. For instance, while Dutch legislation is more stringent with the contaminants found in soil and groundwater (e.g., Dutch list reference values 2013), the Romanian legislation on contamination is lighter (e.g., Ministerial Order (MO) reference values 756/1997 and 621/2014).

### Multiple data source integration in HHRA tools

Integration of multiple data sources in the models, such as the Geographic Information System (GIS) techniques representing the hotspots of the contaminated area and grade of contamination and dispersion, brings a high value to the assessment and supporting the user’s case. Moreover, while integrating diverse functions to the model enhances the capacity of the researchers, policymakers effectively manage the risks with robust data generated and a broader visibility of the CSM (Bień et al. 2005). As a matter of fact, the tools in discussion offer various extra functions sustaining the assessment (Table 5).

**Table 5:** Additional integrated options within the tools discussed (Soltani et al. 2016; Jeffries 2009; Breemen et al. 2020; Larsen 2007; Connor et al. 2001; Cornelis et al. 2022; Spence and Walden 2013; McKone 1993a; McKnight and Finkel 2013; SNPA and ISPRA 2021)

Tools	Human health risks	Ecological risks	Uncertainty and sensitivity analysis	Technologies integrated				
				***GIS***	***Remediation***			***Health effects***
					***Technology***	***Values***	***Cost analysis***	
1 CLEA	Y							
2 CSOIL	Y		Y					
3 JAGG		Y`						
4 RBCA	Y	Y	Y					
5 Risk-net	Y							
6 S-RISC	Y					Y		
7 RISC5 or BP-RISC	Y	Y						
8 CalTOX	Y	Y*	Y	Y**				
9 RECOLAND/REMPET	Y			Y	Y	Y	Y	Y (REMPET only)
10 Caro-plus	Y	Y***	Y				Y	
11 ROME plus	Y							
12 K-RBCA	Y							

^*^Limited application

^**^ARC/INFO analyses

^***^Includes groundwater and surface water

Integrating the contamination area into a map is a challenge, especially if there is multi-component contamination. As stated in the article by Liu et al. (2023), a limited number of investigations have integrated the risk evaluation and its spatial distribution to facilitate a systematic and thorough analysis. For instance, the RECOLAND/REMPET developers have incorporated Bing Maps in the tools on showcasing the contamination level applicable to the Romanian regulation at the site location. CalTOX utilizes GIS software, specifically ARC/INFO and source STATSGO database, to characterize the site feature and generate values for landscape variables. Given this feature of the CalTOX model, it predicts the potential exposure and risk or HI with high accuracy at the sites under the DTSC regulation (Schwalen and Kiefer 1996).

Uncertainty in the risk assessment evaluation is another important aspect for decision making (Dong et al. 2015). In order to facilitate the management of risks associated with exposure to pollutants based on quantitative uncertainty measures, a number of tools such as CalTOX, RBCA, CSOIL, and CARO-PLUS have incorporated common methods for uncertainty analysis, providing confidence intervals based on the model predictions (Dong et al. 2015; McFarland and DeCarlo 2020). The sensitivity analysis provided in the models is directly linked to the initial chemical inventory and input parameter information used (McKone 1993a), (Connor et al. 2001). The benefit of the tools with uncertainty analysis is the possibility of incorporating them into the management and decision-support system, considering the accuracy of the output results (Kelly et al. 2013).

The risk management (RM) decisions regarding contaminated sites typically are represented by guideline values and are taken based on costs, time, and risk reductions achieved (Sorvari and Seppälä 2010). As such, a few tools have adapted RM options for remediation. In particular, CARO-PLUS is focusing on providing cost-effective analysis of the remediation option (McKnight and Finkel 2013). As mandated by Flanders law and site-specific remediation goals, S-RISK offers generic HH-based soil remediation values (Cornelis et al. 2022). RECOLAND/REMPET have introduced a processing date module of optimal remediation method in terms of time, costs, and efficiency based on the human health risks generated in previous modules.

Besides the HHRA, a couple of tools have taken into account in the assessment of Ecological Risks (ERA), which is understood as the contaminated sites affecting plants, animals, and the entire ecosystem, e.g., RISC5, which is designed to calculate both HHRA and ERA from pollutants in the environment (Spence and Walden 2013).

## Recommendations and future perspectives

### Main results obtained within the review

This review paper examined 12 human health risk assessment (HHRA) tools to explore the connections between policy frameworks, cross-sectoral collaboration in land management, and technological advancements in risk assessment. Key concerns related to the models analyzed are highlighted in the context of the reviewed topics.

The main discussion points include:

- HHRA implementation across different countries;
- Legislative frameworks governing tool development;
- Exposure pathways and receptors;
- Pollutants considered;
- Additional data sources integrated into the models.

Based on this review, the key findings are summarized as follows:

- Legislative influence: The legislative context of each country has driven the development of software tools by researchers and private entities to assess risks from contaminated sites to humans and ecosystems.
- Variability in input parameters: The tools reviewed exhibit differences in input parameters, such as exposure durations (EDs), exposure factors (EFs), and acceptable cancer risk (CR) thresholds.
- Exposure pathways: Most tools (11 out of 12) account for soil ingestion and dermal contact, while 10 also incorporate inhalation as an exposure route. Only three models include both on-site and off-site receptors.
- Age categories: Age classifications vary based on national legislation and recent research advancements, though most models primarily categorize receptors as children or adults.
- Contaminant databases: The contaminants covered in each model differ. Some tools focus on conventional site contaminants, while others incorporate complex mixtures (e.g., CARO-PLUS) or emerging “forever chemicals” such as PFAS (e.g., CSOIL and JAGG).
- Additional functionalities: Several tools go beyond basic HHRA modeling. Some integrate GIS-based visualization (e.g., RECOLAND/REMPET), while others include uncertainty analysis (e.g., CARO-PLUS).

## Future research

Based on the findings of this study, several key aspects should be prioritized when developing new risk assessment tools or updating existing ones for contaminated sites. These recommendations focus on identifying the best available techniques to enhance quality and address the gaps identified in the twelve international tools analyzed.

One of the most critical elements is ensuring that future tools are aligned with national legislation while maintaining a user-friendly and intuitive interface. To improve flexibility, it would be beneficial for these tools to integrate threshold values specific to each country while also allowing users to upload alternative datasets or modify legal thresholds according to other regulatory frameworks. Additionally, incorporating an overview of local legislation would optimize decision-making processes for experts working in contaminated site assessment.

Another essential aspect concerns exposure pathways and receptor types. The risk-based approach for contaminated sites relies on the source-pathway-receptor model, yet not all existing tools include a comprehensive range of possible exposure routes. Expanding the list of exposure pathways would improve the applicability of these tools, making them more useful for both public and private entities seeking precise and efficient risk assessments. Currently, most models focus primarily on direct exposure pathways while paying limited attention to indirect routes. Notably, risks associated with the consumption of vegetables and fruits are widely covered, whereas very few tools address potential exposure through meat and dairy products. Among the reviewed models, only CalTOX considers a multi-step exposure scenario, including air, plants, animals, and humans.

Although occupational exposure is considered in all tools, there is a noticeable gap in assessing the risks faced by agricultural workers, particularly in relation to pesticide and fungicide exposure under high-temperature conditions. Additionally, while many models include sensitive receptors such as children and the elderly, they fail to account for individuals with disabilities or pre-existing medical conditions. None of the existing tools assess the risks that contaminants pose to pregnant women, nor do they consider the possibility of placental transfer of harmful substances. Moreover, a bigger attention has to be paid to the children’s receptor category as during early years they are highly vulnerable to external factors. Additional parameters, environmental variables, and field data collection have to be included in the tools to obtain a real case result. Some tools, as is the case of CSOIL or RECOLAND/REMPET, consider children a cumulative age gap between 0 and 6 or 0 and 16; herein, this might result in the underestimation of risk.

Another limitation of current models is their reliance on a fixed exposure pathway assumption over time and space. This simplification often leads to conservative risk estimation, whereas, in reality, individuals are exposed to multiple pathways across different locations. Future advancements should allow for case-specific exposure scenarios that take into account variations in location and mobility. Expanding receptor categories and exposure pathways to include indirect exposure and multiple-location scenarios would significantly improve the accuracy and applicability of risk assessments.

The selection of contaminants in risk assessment tools also requires updates and improvements. In many cases, the chemicals considered are limited to site-specific needs, leading to an incomplete evaluation of potential hazards. For instance, tools such as RECOLAND and REMPET assess only carcinogenic compounds, highlighting the need for an updated contaminant list that includes a broader range of toxic substances. Given that some tools have not been updated in several years, there is a pressing need to reassess the carcinogenic classifications in accordance with the latest EPA regulations. Emerging contaminants, such as PFAS, are not covered in most tools, with the exception of CSOIL and JAGG.

In terms of data source integration, risk assessment tools rely on mathematical models that vary in accuracy depending on the specific national regulations they adhere to. As a result, the risk estimates generated by these models may not always be precise. To improve reliability, it is essential for future tools to incorporate uncertainty analysis throughout the entire risk assessment process. Additionally, integrating GIS-based spatial planning would provide a valuable visual representation of data, aiding in decision-making and site management.

Finally, many of the current models lack robust uncertainty analysis, which can lead to inaccurate risk estimations. To enhance credibility and transparency, a greater level of harmonization across different software tools is needed.

By addressing these key areas, future risk assessment tools can provide more accurate, flexible, and comprehensive evaluations of contaminated sites.

## Data Availability

No new data was created or analyzed in this study. Data sharing is not applicable to this article.

## Nomenclature

US EPA: United States Environmental Protection Agency
DEPA: Danish Environmental Protection Agency
APAT: Italian Agency for Environmental Protection and Technical Services
MATTM: Ministry of the Environment and Protection of Land and Sea of Italy
INAIL: National Institute for Insurance against Accidents at Work in Italy
DTSC: Department of Toxic Substances Control
IRIS: Integrated Risk Information System
VMR: Regional Knowledge Center for Environmental Resources
WHO: World Health Organization
FAO: Food and Agriculture Organization
CR: Carcinogenic risk
CSM: Conceptual site model
EP: Exposure pathway
EF: Exposure frequency
ED: Exposure duration
HI: Hazard index
HHRA: Human health risk assessment
ERA: Ecological risk assessment
EPA: Environmental Protection Agency
ASTM: American Society for Testing and Materials
RBCA: Risk-based corrective action
PFAS: Per- and polyfluoroalkyl substances
BTEX: Benzene, toluene, ethylene, and xylene
PAH: Polycyclic aromatic hydrocarbons
PCB: Polychlorinated biphenyl
DNAPL: Dense non-aqueous phase liquid
LNAPL: Light non-aqueous phase liquid
VOC: Volatile organic compounds
BPA: Bisphenol A
DEHP: Diethylhexyl phthalate
GIS: Geographic information system
RM: Risk management
